# Intra-carotid arterial transfusion of circulatory-derived autologous endothelial progenitor cells in rodent after ischemic stroke—evaluating the impact of therapeutic time points on prognostic outcomes

**DOI:** 10.1186/s13287-020-01739-y

**Published:** 2020-06-05

**Authors:** Kun-Chen Lin, Han-Tan Chai, Kuan-Hung Chen, Pei-Hsun Sung, John Y. Chiang, Pei-Lin Shao, Chi-Ruei Huang, Yi-Chen Li, Sheung-Fat Ko, Hon-Kan Yip

**Affiliations:** 1 Department of Anesthesiology, Kaohsiung Chang Gung Memorial Hospital and Chang Gung University College of Medicine, Kaohsiung, Taiwan; 2 Division of Cardiology, Department of Internal Medicine, Kaohsiung Chang Gung Memorial Hospital and Chang Gung University, College of Medicine, Kaohsiung, Taiwan; 3 Center for Shockwave Medicine and Tissue Engineering, Kaohsiung Chang Gung Memorial Hospital, Kaohsiung, Taiwan; 4 Department of Computer Science and Engineering, National Sun Yat-Sen University, Kaohsiung, Taiwan; 5 Department of Healthcare Administration and Medical Informatics, Kaohsiung Medical University, Kaohsiung, Taiwan; 6 Department of Nursing, Asia University, Taichung, Taiwan; 7 Department of Radiology, Kaohsiung Chang Gung Memorial Hospital and Chang Gung University College of Medicine, Kaohsiung, Taiwan; 8 Institute for Translational Research in Biomedicine, Kaohsiung Chang Gung Memorial Hospital, Kaohsiung, Taiwan; 9 Department of Medical Research, China Medical University Hospital, China Medical University, Taichung, Taiwan; 10 Division of Cardiology, Department of Internal Medicine, Xiamen Chang Gung Hospital, Xiamen, Fujian China

**Keywords:** Ischemic stroke, Endothelial progenitor cells, Angiogenesis, Neurological function

## Abstract

**Background:**

This study tested the optimal time point for left intra-carotid arterial (LICA) administration of circulatory-derived autologous endothelial progenitor cells (EPCs) for improving the outcome in rat after acute ischemic stroke (IS).

**Methods and results:**

Adult male SD rats (*n* = 70) were equally categorized into group 1 (sham-operated control), group 2 (IS), group 3 (IS+EPCs/1.2 × 10^6^ cells/by LICA administration 3 h after IS), group 4 (IS+EPCs/LICA administration post-day-3 IS), group 5 (IS+EPCs/LICA administration post-day-7 IS), group 6 (IS+EPCs/LICA administration post-day-14 IS), and group 7 (IS+EPCs/LICA administration post-day-28 IS). The brain infarct volume (BIV) (at day 60/MRI) was lowest in group 1, highest in group 2, and significantly progressively increased from groups 3 to 7, whereas among the IS animals, the neurological function was significantly preserved in groups 3 to 6 than in groups 2 and 7 post-day-60 IS (all *P* < 0.0001). By day 60, the endothelial cell markers at protein and cellular levels and number of small vessels exhibited an opposite pattern of BIV among the groups (all *P* < 0.0001). The protein and cellular levels of inflammation, and protein levels of oxidative stress, autophagy, and apoptosis were highest in group 2, lowest in group 1, and progressively increased from groups 3 to 7 (all *P* < 0.0001). The angiogenesis biomarkers at protein and cellular levels were significantly progressively increased from groups 1 to 3, then significantly progressively decreased from groups 4 to 7 (all *P* < 0.0001).

**Conclusion:**

Early EPC administration provided better benefits on improving functional/image/molecular-cellular outcomes after acute IS in rat.

## Introduction

Although divergent etiologies cause stroke, atherosclerotic intracranial arterial stenosis/occlusion remains one of the common causes of ischemic stroke (IS) worldwide [1–6] and is associated with a high risk of frequently recurrent IS once IS developed [7–9]. Surprisingly, while the epidemiology, etiologies, mechanisms, classification, and prognostic outcomes of IS have been widely investigated for several decades, a safe and effective treatment strategy for the majority of patients after acute IS has not been fully developed [10–14]. Hence, finding a safe and effective therapeutic option for IS patients is an important issue.

Our previous studies have shown that the circulating level of endothelial progenitor cells (EPCs) was notably frequently increased in patients after acute IS [15, 16], suggesting acute IS event would stimulate the EPC mobilization from bone marrow to circulation. Additionally, our studies [15, 16] have further identified that an increase in circulating level of EPCs was strongly associated with favorable clinical outcomes after IS [15, 16]. Importantly, these findings demonstrated that circulating level of EPCs can serve as a useful biomarker for stratification of IS patients into high- and low-risk groups with respect to future outcomes [15, 16]. Based on the findings from our studies [15, 16], we subsequently performed an acute IS model in rodent and treated by intra-carotid artery administration of circulatory-derived autologous EPCs [17]. Intriguingly, the results revealed that this therapy significantly reduced the brain infarct size and improved neurological dysfunction after acute IS in rodent mainly through enhancement of angiogenesis and neurogenesis and reduction of inflammation, oxidative stress, and cellular apoptosis [17]. The results of these studies [15–17] encouraged us to perform a phase I clinical trial of intra-carotid artery transfusion of circulatory-derived autologous EPCs into brain infarct area in old IS stroke patients [18]. The results of our study displayed that EPC therapy was safe. However, the neurological and neuro-psychiatric functions as well as the brain perfusion status were found to have only minor improvement in those patients [18]. The results of these aforementioned unmet needs from our study [18] raise the consideration of a large randomized placebo-controlled trial to test the safety and efficacy of EPC therapy for patients after acute IS. In light of the above observation, an animal model of IS study, before applying for a clinical trial, focusing on the optimal time point for administration of EPCs that would offer the greatest benefit and the minimal side effect is required. Accordingly, this study using a rat model of acute IS tried to find out the best time point of circulatory-derived autologous EPC administration for reducing the brain infarct volume (BIV)/brain infarct area (BIA) and improving the neurological outcome in IS rat.

## Materials and methods

### Procedure and protocol of acute IS induction

The protocol and procedure for the experimental model of acute IS have been described in our previous studies [17, 19]. In detail, each animal was anesthetized by 2% inhalational isoflurane in a supine position on a warming pad (37 °C). After exposure of the left common carotid artery (LCCA) through a transverse neck incision, a small arteriotomy was performed on the LCCA through which silicon rubber-coated monofilament (filament size 4–0, diameter 0.185 mm, length 30 mm; Co Ltd. AMBO) was carefully advanced into the distal left internal carotid artery for occlusion of the left middle cerebral artery, causing brain ischemia and infarction of the corresponding territory area. The nylon monofilament was removed 50 min after occlusion, followed by closure of the muscle and skin in layers. The animals were then cared for in a portable animal intensive care unit (ThermoCare®) with food and water for 24 h.

### Corner test for assessment of neurological function prior to and after IS induction

The sensorimotor functional test (corner test) was conducted for each rat at baseline and on days 0, 3, 7, 14, 28, and 60 after acute IS induction as we previously described [17, 19]. In detail, the rat could walk through a tunnel and then turn into a 60° corner. To exit the corner, the rat could turn either left or right. The results were recorded by a technician blinded to the study design. This test was repeated 10 to 15 times with at least 30 s between each trial. We recorded the number of right and left turns from 10 successful trials for each animal and used the results for statistical analysis. All 10 animals in each group went through this neurological functional test.

### Animal grouping and treatment protocol

Animals were housed in an Association for Assessment and Accreditation of Laboratory Animal Care International-approved animal facility in our hospital, with controlled temperature and light cycles (24 °C and 12/12 light/dark cycle). Pathogen-free, adult male Sprague-Dawley (SD) rats (*n* = 70) weighing around 300–325 g (Charles River Technology, BioLASCO Taiwan Co. Ltd., Taiwan) were utilized in the present study. Among the animals, the sham-operated control (SC) was first randomized. After acute IS induction procedure, the animals were further randomized into acute ischemic stroke (IS), acute IS + EPCs (1.2 × 10^6^ cells by left intra-carotid arterial (ICA) administration at 3 h after IS) (EPC^3h^), acute IS + EPCs (1.2 × 10^6^ cells by left ICA administration at day 3 after IS) (EPC^3d^), acute IS + EPCs (1.2 × 10^6^ cells by left ICA administration at day 7 after IS) (EPC^7d^), acute IS + EPCs (1.2 × 10^6^ cells by left ICA administration at day 14 after IS) (EPC^14d^), and acute IS + EPCs (1.2 × 10^6^ cells by left ICA administration at day 28 after IS (EPC^28d^), respectively. At 1.2 h after removing the silicon rubber-coated monofilament, the EPCs were slowly and carefully transfused into the LCCA by a 27# needle.

The number of operated-related (i.e., included IS induction and EPC administration) dead animals in SC, IS, EPC^3h^, EPC^3d^, EPC^7d^, EPC^14d^, and EPC^28d^ were 0, 4, 3, 5, 4, 3, and 5, respectively. On the other hand, no any animal was excluded due to unsuccessful induction.

The dosage of EPCs administered in the present study was based on our previous report [17]. For the molecular-cellular examination, six animals in each group were utilized.

Ibuprofen (15 mg/kg/day) dissolved in drinking water was orally administered for the pain management for the animals for 3 to 7 days after the IS-inducing procedure.

Animals in each group were euthanized by day 60 after IS induction. Prior to euthanization, the animals were anesthetized by 5% of isoflurane mixed with 100% of oxygen 3 L/min, followed by drawing the blood 10 mL from abdominal aorta to induce hypovolemic shock and death. Finally, the wound was sutured after the brain specimen was harvested from each animal.

### Peripheral blood was collected and cultured for EPCs

The procedure and protocol for EPC isolation and culture were based on our previous reports [17] with some modifications. In brief, animals in groups EPC^3h^, EPC^3d^, EPC^7d^, and EPC^14d^ were anesthetized with inhalational 2.0% isoflurane, i.e., by day 21 at EPC^3h^, by day 18 at EPC^3d^, by day 14 at EPC^d7^, and by day 7 at EPC^14d^ prior to IS induction and by day 7 after IS induction at EPC^28d^ to collect peripheral blood for culturing EPCs (refer to the supplementary Fig. 1). Isolated mononuclear cells from peripheral blood were cultured in a 100-mm diameter dish with 10 mL DMEM culture medium containing 10% FBS for 21 days. Based on our previous method [17], flow cytometric analysis was performed for identification of cellular characteristics (i.e., EPC surface markers) after cell-labeling with appropriate antibodies on day 21 of cell cultivation prior to cell therapy (i.e., EPCs were transfused into LCCA on day 21 after cell culture) (refer to the supplementary Fig. 2).

Rats in groups 1 and 2 were anesthetized with inhalational 2.0% isoflurane and only received an identical procedure of peripheral blood sampling without further culture.

### Procedure and protocol of brain magnetic resonance imaging (MRI) study

The procedure and protocol have been described in our previous report [17]. Magnetic resonance imaging (MRI) was performed at day 60 and after acute IS induction. Briefly, during MRI measurements, mice were anesthetized by 2% inhalational isoflurane with room air and placed in an MRI-compatible holder (Biospec 94/20, Bruker, Ettingen, Germany). Rectal temperature and respiration were monitored throughout the procedure to ensure normal physiological conditions were maintained. MRI data were collected using a Varian 9.4-T animal scanner (Biospec 94/20, Bruker, Ettingen, Germany) with a rat surface array. The MRI protocol consisted of 40 T2-weighted images. Forty continuous slice locations were imaged with a field of view of 30 mm × 30 mm and an acquisition matrix dimension of 256 × 256 and slice thickness of 0.5 mm. The repetition time (TR) and echo time (TE) for each fast spin-echo volume were 4200 ms and 30 ms, respectively. A custom software, ImageJ (1.43i, NIH, USA), was used to process the region of interest (ROI). Planimetric measurements of images from MRI T2 were performed to calculate the stroke volumes of cortex. For brain MRI examination, four randomized animals in each group were utilized. The animals were euthanized on day 60 after brain MRI examination and the brain tissues were harvested for individual study.

### Immunofluorescent (IF) staining of brain specimens

The procedure and protocol for IF staining have been reported in our previous studies [17–19]. For IF staining, rehydrated paraffin sections were first treated with 3% H_2_O_2_ for 30 min and incubated with Immuno-Block reagent (BioSB, Santa Barbara, CA, USA) for 30 min at room temperature. Sections were then incubated with primary antibodies specifically against CD31 (1:100, Bio-Rad), von Willebrand factor (vWF) (1:200, Merck Millipore), NeuN (1:1000, Millipore, Billerica, MA, USA), stromal cell-derived factor [(SDF)-1α (1:100, Santa Cruz Biotechnology), vascular endothelial growth factor (VEGF) (1:400, Abcam), CXCR4 (1:200, Thermo Fisher Scientific), microglial cell (1:100, Abcam, Cambridge, UK), CD68 (1:100,Abcam, Cambridge, UK), and glial fibrillary acidic protein (GFAP) (1:500, Dako) at 4 °C overnight. Alexa Fluor488, Alexa Fluor568, or Alexa Fluor594-conjugated goat anti-mouse or rabbit IgG were used to localize signals. Sections were finally counterstained with DAPI and observed with a fluorescent microscope equipped with epifluorescence (Olympus IX-40).

Three brain sections were analyzed for each rat. For quantification, three randomly selected high-power fields (HPFs; × 400 for IF study) were analyzed in each section. The mean number of positively stained cells per HPF for each animal was then determined by summation of all numbers divided by 9.

### Western blot analysis of brain specimens

The procedure and protocol for Western blot have been reported in our previous studies [17–19]. In details, equal amounts (50 μg) of protein extracts were loaded and separated by SDS-PAGE using 12% acrylamide gradients. After electrophoresis, the separated proteins were transferred electrophoretically to a polyvinylidene difluoride (PVDF) membrane (Amersham Biosciences, Amersham, UK). Nonspecific sites were blocked by incubation of the membrane in blocking buffer [5% nonfat dry milk in T-TBS (TBS containing 0.05% Tween 20)] overnight. The membranes were incubated with monoclonal antibodies against CXCR4 (1:1000, Abcam), SDF-1α (1:1000, Cell Signalimg, Danvers, MA, USA), VEGF (1:1000, Abcam), CD31 (1:1000, Abcam), von Willebrand factor (vWF) (1:1000, Abcam), NOX-1 (1:1500, Sigma-Aldrich), NOX-2 (1:500, Sigma-Aldrich), matrix metalloproteinase (MMP)-9 (1:3000, Abcam), tumor necrosis factor alpha (TNF-α; 1:1000, Cell Signaling), nuclear factor (NF)-κB (1:1000, Abcam), interleukin (IL)-1ß (1:1000, Cell Signaling), caspase 3 (1:3000, Abcam), mitochondrial-Bax (1:1000, Abcam), beclin-1 (1:1000, Cell Signaling), Atg5 (1:1000, Cell Signaling), LCB3-II (1:2000, Abcam), and LCB3-I (1:2000, Abcam) for 1 h at room temperature. Horseradish peroxidase-conjugated anti-rabbit or anti-mouse immunoglobulin IgG (1:2000, Cell Signaling) was used as a second antibody for 1 h at room temperature. The washing procedure was repeated eight times within 1 h, and immunoreactive bands were visualized by enhanced chemiluminescence (ECL; Amersham Biosciences) and exposed to Medical X-ray film (FUJI). For quantitation, ECL signals were digitized using Labwork software (UVP, Waltham, MA, USA). A standard control sample was loaded on each gel.

### Vessel density in peri-infarct area

Immunohistochemistry (IHC) staining of small blood vessels was performed with α-SMA (1:400) as primary antibody at room temperature for 1 h, followed by washing with PBS three times. Ten minutes after the addition of anti-mouse-HRP conjugated secondary antibody, the tissue sections were washed with PBS three times. Then 3,3′ diaminobenzidine (DAB) (0.7 g/tablet) (Sigma-Aldrich) was added, followed by washing with PBS three times after 1 min. Finally, hematoxylin was added as a counter-stain for nuclei, followed by washing twice with PBS after 1 min. Three brain sections were analyzed in each rat. For quantification, three randomly selected HPFs (× 200) were analyzed in each section. The mean number per HPF for each animal was then determined by summation of all numbers divided by 9.

### Statistical analyses

Quantitative continuous data are expressed as mean ± SD. Statistical analysis was performed for continuous variables among groups by one-way ANOVA followed by the Bonferroni multiple-comparison post hoc test for the between-group comparison. All analyses were conducted using SAS statistical software for Windows version 8.2 (SAS institute, Cary, NC, USA). A probability value < 0.05 was considered statistically significant.

## Results

### The brain infarct volume (BIV) at day 60 after IS and the time courses of corner test after acute IS (Figs. 1 and 2)

**Fig. 1:**
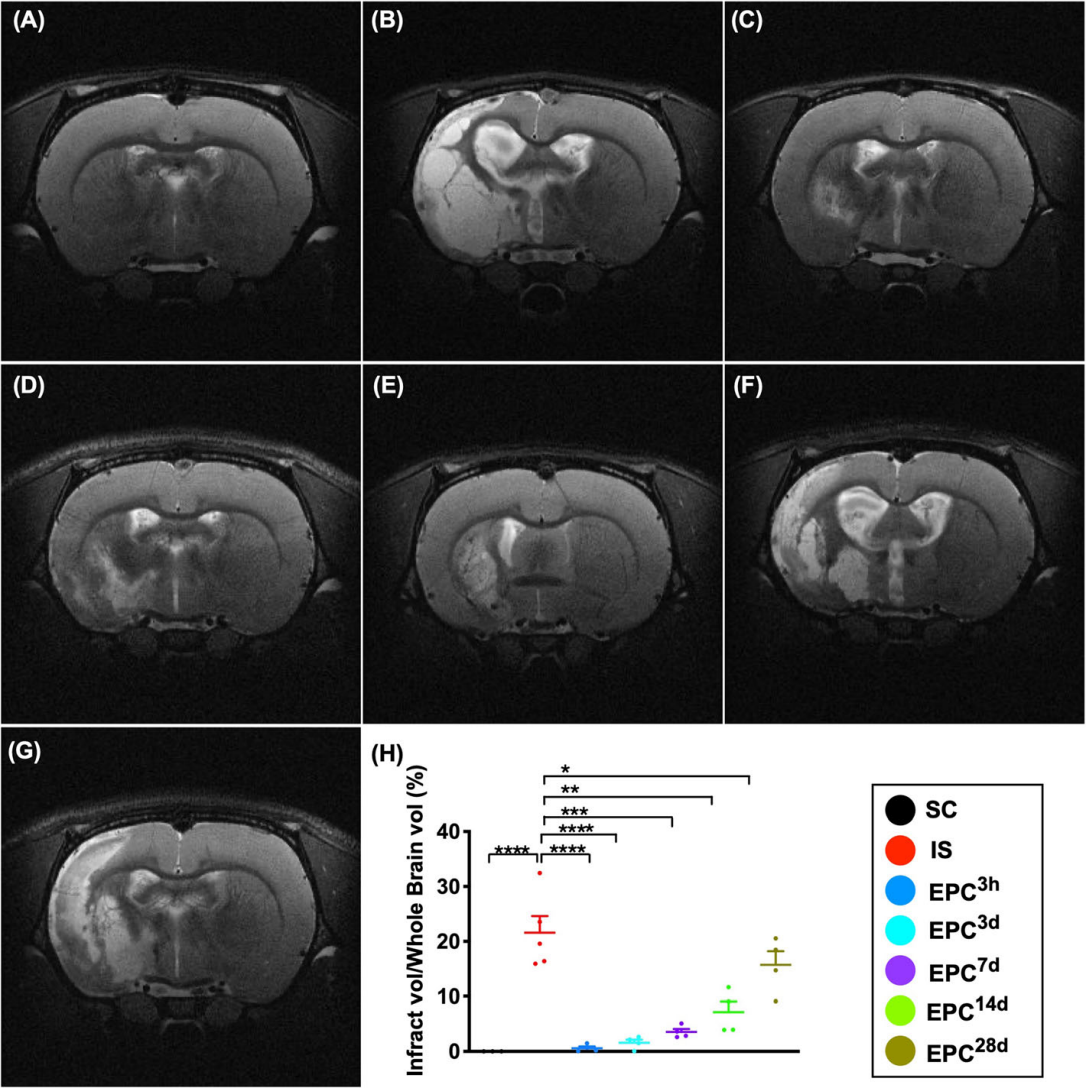
The brain infarct volume (BIV) at day 60 after acute IS. **a**–**g** Illustrating the brain magnetic resonance imaging (MRI) study for identification of brain infarct zone (whitish color)/BIV. **h** Analytical result of BIV (*n* = 4), *P < 0.05, **P < 0.01, ***P < 0.001, ****P < 0.0001 vs. IS group. All statistical analyses were performed by one-way ANOVA, followed by Bonferroni multiple-comparison post hoc test (*n* = 4 for each group). Vol = volume. LCCA = left common carotid artery. SC = sham-operated control; IS = ischemic stroke (IS); EPC^3h^ = EPC administration from LCCA at 3 h after acute IS; EPC^3d^ = EPC administration from LCCA at day 3 after acute IS; EPC^7d^ = EPC administration from LCCA at day 7 after acute IS; EPC^14d^ = EPC administration from LCCA at day 14 after acute IS; EPC^28d^ = EPC administration from LCCA at day 28 after acute IS

At day 60 after acute IS, the brain MRI demonstrated that the BIV was lowest in SC, highest in IS, and significantly and progressively increased from EPC^3h^, EPC^3d^, EPC^7d^, EPC^14d^, to EPC^28d^ (Fig. 1).

**Fig. 2:**
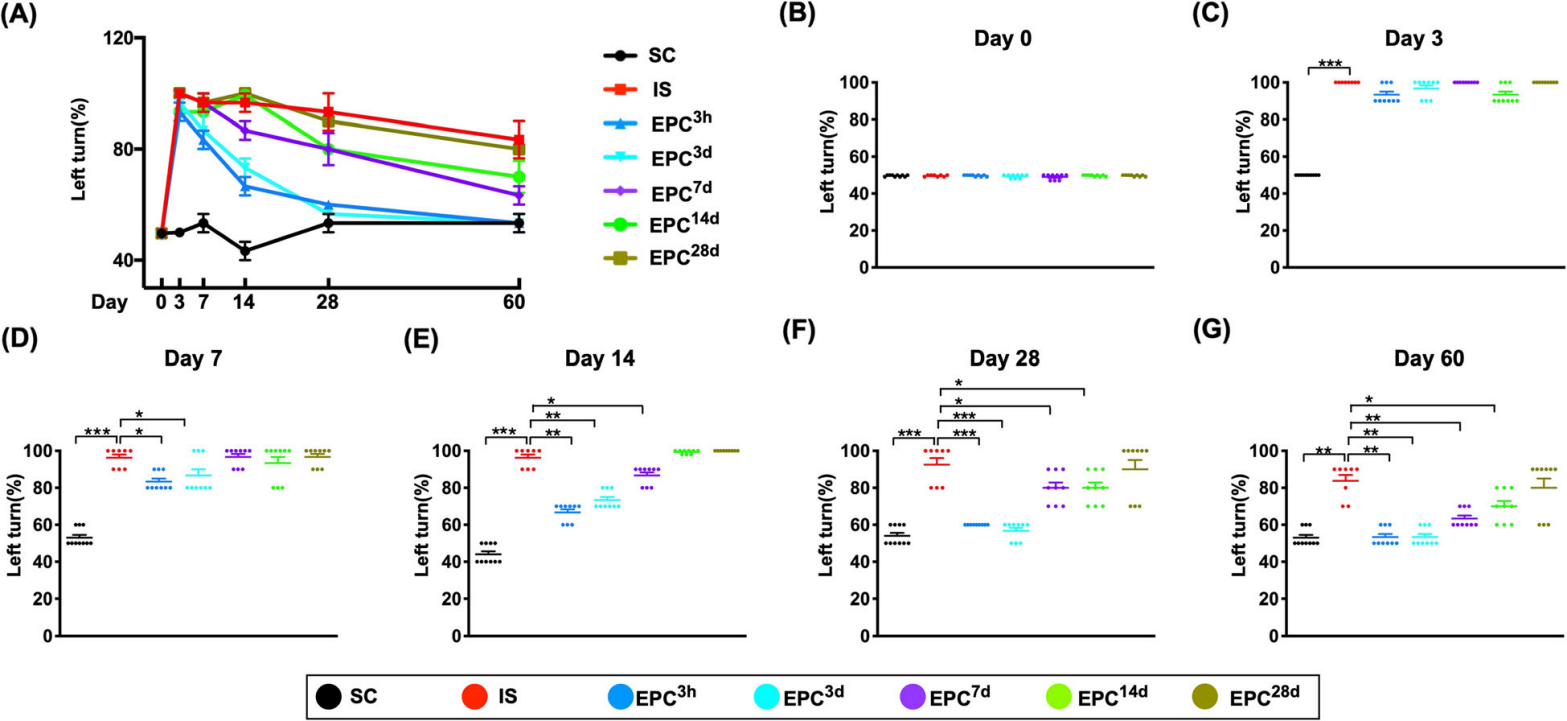
Time courses of corner test for assessment of neurological function at different time points after acute IS. **a** Graphical illustration of time points of corner test for determining the neurological function prior to and after acute IS among the seven groups. **b** At day 0, *P* > 0.5. **c** At day 3, ***P < 0.001 vs. SC. **d** At day 7, *P < 0.05, **P < 0.01, ***P < 0.001 vs. IS. **e** At day 14, * < 0.05, **P < 0.01, ***P < 0.001 vs. IS. **f** At day 28, *P < 0.05, ***P < 0.001 vs. IS. **g** At day 60, *P < 0.05, **P < 0.01. All statistical analyses were performed by one-way ANOVA, followed by Bonferroni multiple-comparison post hoc test (*n* = 10 for each group). SC = sham-operated control; IS = ischemic stroke (IS); EPC^3h^ = EPC administration from LCCA at 3 h after acute IS; EPC^3d^ = EPC administration from LCCA at day 3 after acute IS; EPC^7d^ = EPC administration from LCCA at day 7 after acute IS; EPC^14d^ = EPC administration from LCCA at day 14 after acute IS; EPC^28d^ = EPC administration from LCCA at day 28 after acute IS

The neurological function did not differ at baseline among seven groups. However, at day 3 after acute IS, the neurological function was significantly impaired in all the IS groups regardless with or without treatment than in SC, but it showed no difference among the six IS groups (Fig. 2).

On the other hand, at day 7 after IS, this parameter was significantly improved in EPC^3h^ and EPC^3d^ than in IS, EPC^7d^ to EPC^28d^, but it did not differ between groups EPC^3h^ and EPC^3d^ or among IS, EPC^7d^, EPC^14d^, and EPC^28d^ (Fig. 2).

Additionally, at day 14 after IS, the neurological defect was lowest in SC, highest in IS, EPC^14d^ and EPC^28d^, and significantly higher in EPC^7d^ than in EPC^3h^ and EPC^3d^, but it showed no difference between EPC^3h^ and EPC^3d^ or among IS, EPC^14d^, and EPC^28d^ (Fig. 2).

Furthermore, by day 28 after IS, the neurological defect was significantly lower in SC, EPC^3h^, and EPC^3d^ than in other groups and significantly lower in EPC^7d^ and EPC^14d^ than in IS and EPC^28d^, but it showed no difference among SC, EPC^3h^, and EPC^3d^, between EPC^7d^ and EPC^14d^, or between IS and EPC^28d^ (Fig. 2).

Finally, at day 60 after IS, the neurological defect was lowest in SC, EPC^3h^, and EPC^3d^, highest in IS and EPC^28d^, and significantly lower in EPC^d7^ than in EPC^d14^, but it did not differ among the SC, EPC^3h^, and EPC^3d^ or between IS and EPC^28d^ (Fig. 2).

### The expressions of angiogenesis biomarkers in protein and cellular levels in BIA at day 60 after acute IS (Figs. 3, 4, 5, and 6)

**Fig. 3:**
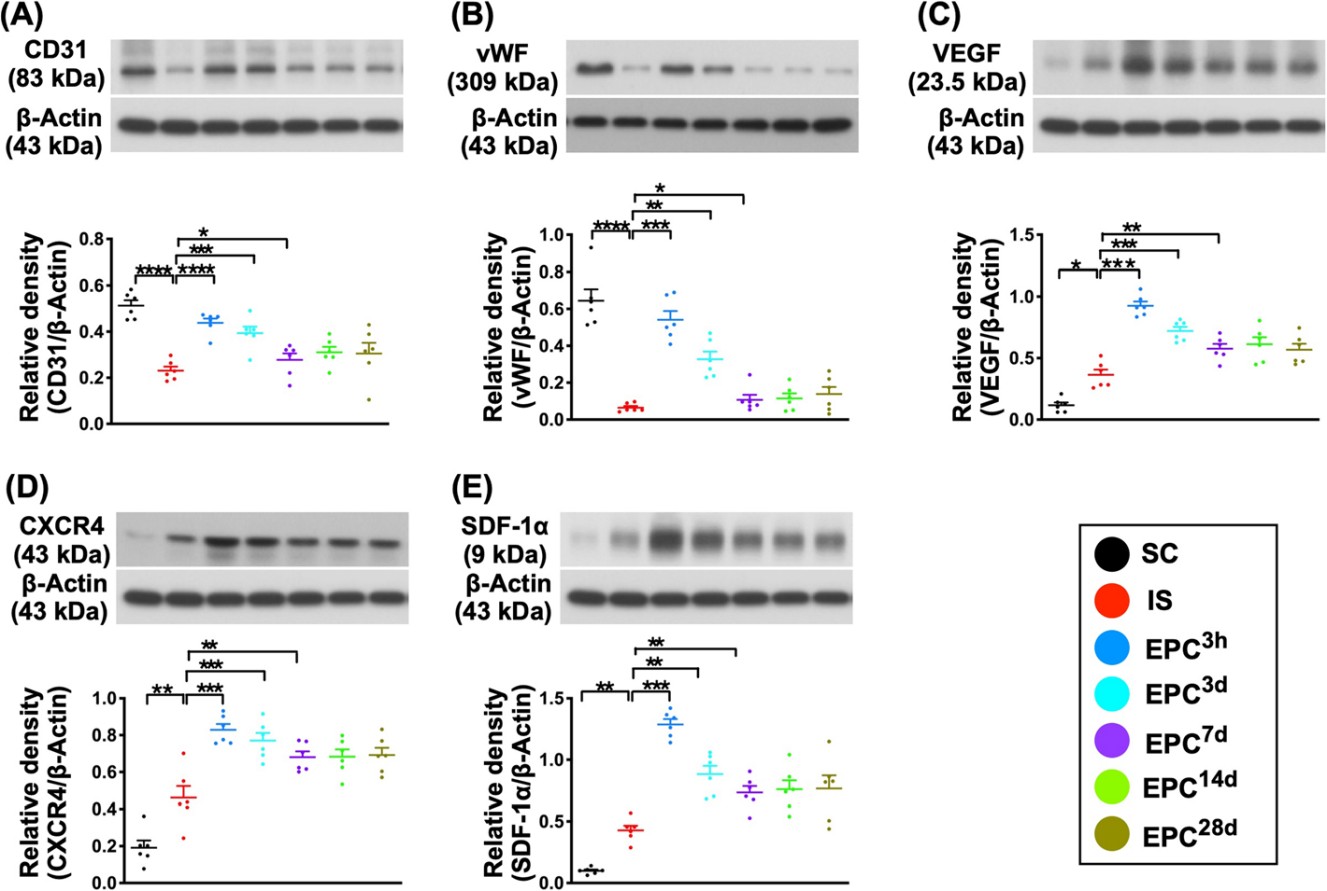
Protein expressions of angiogenesis biomarkers in brain infarct zone by day 60 after acute IS. **a** Protein expression of CD31, *P < 0.05, ***P < 0.001, ****P < 0.0001 vs. IS. **b** Protein expression of von Willebrand factor (vWF), *P < 0.05, **P < 0.01, ***P < 0.001, ****P < 0.0001 vs. IS. **c** Protein expression of vascular endothelial growth factor (VEGF), *P < 0.05, **P < 0.01, ***P < 0.001, ****P < 0.0001 vs. IS. **d** Protein expression of CXCR4, **P < 0.01, ***P < 0.001, ****P < 0.0001 vs. IS. **e** Protein expression of stromal cell-derived growth factor (SDF)-1α, **P < 0.01, ***P < 0.001, ****P < 0.0001 vs. IS. All statistical analyses were performed by one-way ANOVA, followed by Bonferroni multiple-comparison post hoc test (*n* = 6 for each group). LCCA = left common carotid artery. SC = sham-operated control; IS = ischemic stroke (IS); EPC^3h^ = EPC administration from LCCA at 3 h after acute IS; EPC^3d^ = EPC administration from LCCA at day 3 after acute IS; EPC^7d^ = EPC administration from LCCA at day 7 after acute IS; EPC^14d^ = EPC administration from LCCA at day 14 after acute IS; EPC^28d^ = EPC administration from LCCA at day 28 after acute IS

First, we performed the Western blotting for identifying the protein level of angiogenesis biomarkers. The results demonstrated that the protein expressions of CD31 and vWF, two indicators of endothelial functional integrity, were highest in SC; lowest in IS; significantly higher in EPC^3h^ and EPC^3d^ than in EPC^7d^, EPC^14d^, and EPC^28d^; and significantly higher in EPC^3h^ than in EPC^3d^, but they did not differ among the EPC^7d^, EPC^14d^, and EPC^28d^ (Fig. 3). Additionally, the protein expressions of VEGF, CXCR4, and SDF-1α, three indices of angiogenesis factors, were lowest in SC and highest in EPC^3h^, significantly higher in EPC^3d^ than in IS, and EPC^7d^ to EPC^28d^ and significantly higher in EPC^7d^ to EPC^28d^ than in IS, but they showed no difference among the groups EPC^7d^ to EPC^28d^ (Fig. 3).

**Fig. 4:**
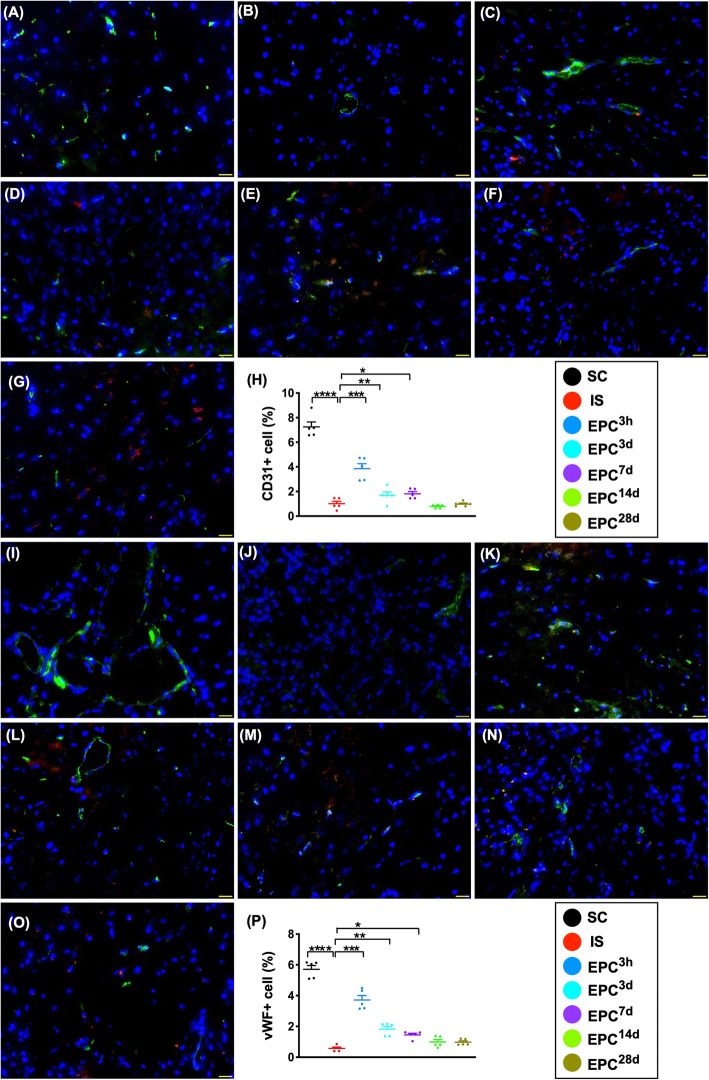
Expressions of endothelial cell biomarkers in brain infarct zone at day 60 after acute IS. **a**–**g** Illustrating immunofluorescent (IF) microscopic finding (× 400) for identification of the expression of CD31+ cells (green color). Red color in **c** to **g** indicated the dye-stained implanted ECPs. **h** Analytical result of number of CD31+ cells, *P < 0.05, **P < 0.01, ***P < 0.001, ****P < 0.0001 vs. IS. **i**–**o** Illustrating IF microscopic finding (× 400) for identification of the expression of von Willebrand factor (vWF) + cells (green color). Red color in **k** to **o** indicates the dye-stained implanted ECPs. **p** Analytical result of number of vWF+ cells, *P < 0.05, **P < 0.01, ***P < 0.001, ****P < 0.0001 vs. IS. Scale bars in lower right corner represent 20 μm. All statistical analyses were performed by one-way ANOVA, followed by Bonferroni multiple-comparison post hoc test (*n* = 6 for each group). LCCA = left common carotid artery. SC = sham-operated control; IS = ischemic stroke (IS); EPC^3h^ = EPC administration from LCCA at 3 h after acute IS; EPC^3d^ = EPC administration from LCCA at day 3 after acute IS; EPC^7d^ = EPC administration from LCCA at day 7 after acute IS; EPC^14d^ = EPC administration from LCCA at day 14 after acute IS; EPC^28d^ = EPC administration from LCCA at day 28 after acute IS

**Fig. 5:**
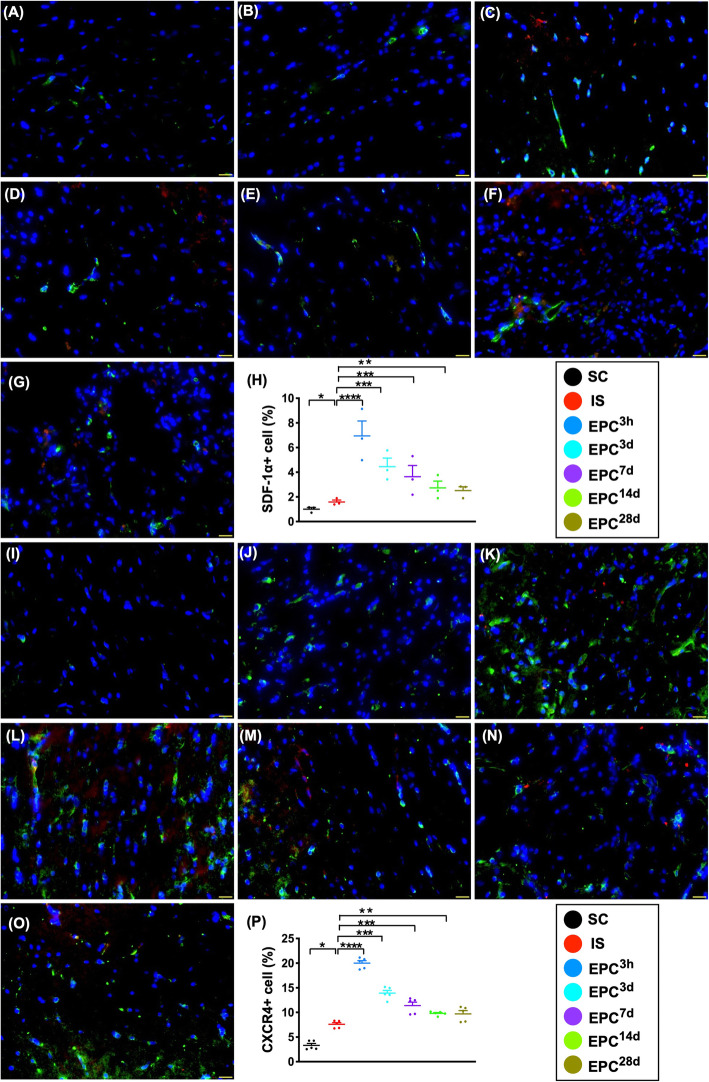
Cellular expressions of angiogenesis factors in brain infarct zone at day 60 after acute IS. **a**–**g** Illustrating immunofluorescent (IF) microscopic finding (× 400) for identification of the expression of stromal cell-derived growth factor (SDF)-1α+ cells (green color). Red color in **c** to **g** indicated the dye-stained implanted ECPs. **h** Analytical result of number of SDF-1α+ cells, *P < 0.05, **P < 0.01, ***P < 0.001, ****P < 0.0001 vs. IS. **i**–**o** Illustrating IF microscopic finding (× 400) for identification of the expression of CXCR4+ cells (green color). Red color in **k** to **o** indicates the dye-stained implanted ECPs. **p** Analytical result of number of CXCR4+ cells, *P < 0.05, **P < 0.01, ***P < 0.001, ****P < 0.0001 vs. IS. Scale bars in lower right corner represent 20 μm. All statistical analyses were performed by one-way ANOVA, followed by Bonferroni multiple-comparison post hoc test (*n* = 6 for each group). LCCA = left common carotid artery. SC = sham-operated control; IS = ischemic stroke (IS); EPC^3h^ = EPC administration from LCCA at 3 h after acute IS; EPC^3d^ = EPC administration from LCCA at day 3 after acute IS; EPC^7d^ = EPC administration from LCCA at day 7 after acute IS; EPC^14d^ = EPC administration from LCCA at day 14 after acute IS; EPC^28d^ = EPC administration from LCCA at day 28 after acute IS

**Fig. 6:**
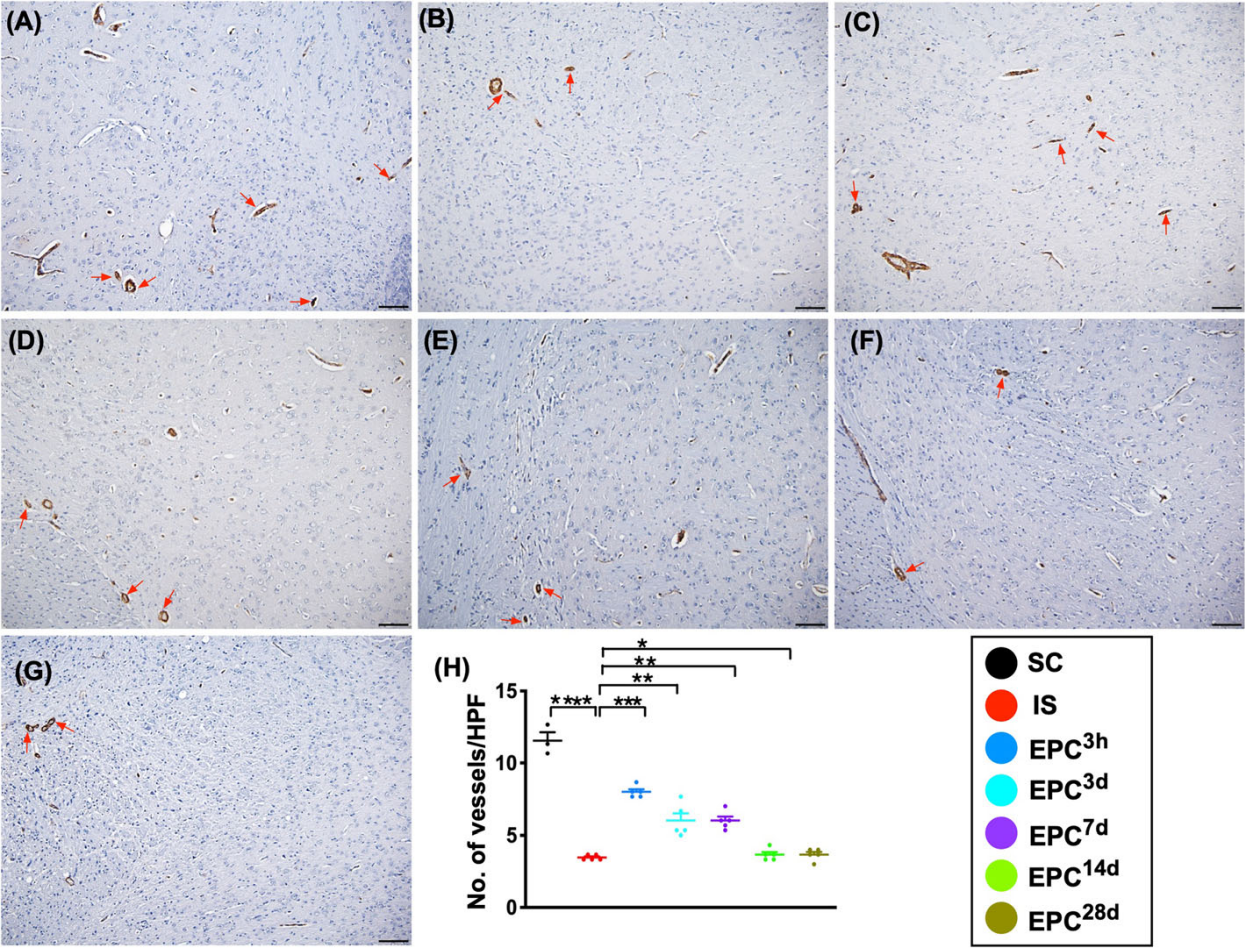
Expression of small vessel density in brain infarct zone at day 60 after acute IS**. a**–**g** Illustrating the microscopic finding (× 100) of α-smooth muscle (SMA) staining for identifying the expression of small vessels (i.e., diameter ≤ 25.0 μm) (red arrows). **h** Analytical result of number of small vessels, *P < 0.05, **P < 0.01, ***P < 0.001, ****P < 0.0001 vs. IS. Scale bars in the lower right corner represent 100 μm. All statistical analyses were performed by one-way ANOVA, followed by Bonferroni multiple-comparison post hoc test (*n* = 6 for each group). LCCA = left common carotid artery. SC = sham-operated control; IS = ischemic stroke (IS); EPC^3h^ = EPC administration from LCCA at 3 h after acute IS; EPC^3d^ = EPC administration from LCCA at day 3 after acute IS; EPC^7d^ = EPC administration from LCCA at day 7 after acute IS; EPC^14d^ = EPC administration from LCCA at day 14 after acute IS; EPC^28d^ = EPC administration from LCCA at day 28 after acute IS

Next, we performed IF microscope for assessment of endothelial cell surface markers. The results displayed that the cellular expressions of CD31 and vWF were highest in SC, lowest in IS, and significantly progressively reduced from EPC^3h^ to EPC^28d^, but they showed no difference between the EPC^14d^ and EPC^28d^ (Fig. 4).

Additionally, the cellular expressions of SDF-1α and CXCR4 were highest in EPC^3h^; lowest in SC; significantly higher in EPC^3d^ than in IS, EPC^7d^, to EPC^28d^; significantly higher in EPC^7d^ to IS, EPC^14d^, and EPC^28d^; and significantly higher in EPC^14d^ and EPC^28d^ than in IS, but they were similar between EPC^14d^ and EPC^28d^, suggesting an intrinsic response to ischemic stimulation that was significantly upregulated by EPC treatment (Fig. 5).

To elucidate the angiogenesis/neovascularization in the peri-infarct region, we performed IHC staining (i.e., α-SMA stain). As expected, the number of small vessel (i.e., diameter ≤ 25 μM) exhibited a similar pattern to that of the protein levels of CD31 and vWF among the groups (Fig. 6).

### The protein expressions of inflammatory, oxidative stress, and autophagic and apoptotic biomarkers in BIA at day 60 after acute IS (Figs. 7 and 8)

**Fig. 7:**
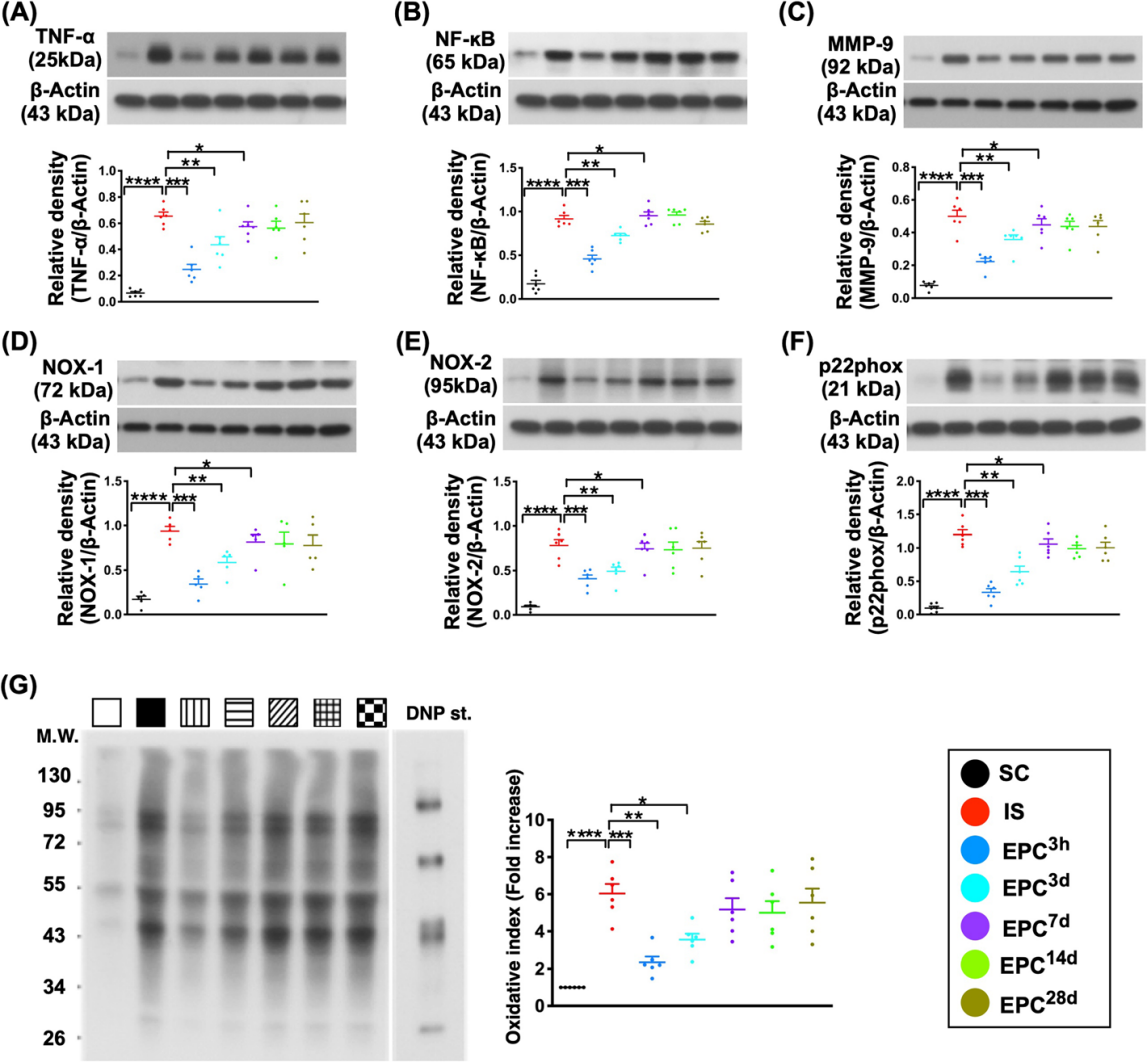
Protein expressions of inflammatory and oxidative stress biomarkers in brain infarct zone at day 60 after acute IS. **a** Protein expression of tumor necrosis factor (TNF-α), *P < 0.05, **P < 0.01, ***P < 0.001, ****P < 0.0001 vs. IS. **b** Protein expression of phosphorylated nuclear factor (p-NF)-κB, *P < 0.05, **P < 0.01, ***P < 0.001, ****P < 0.0001 vs. IS. **c** Protein expression of matrix metalloproteinase (MMP)-9, *P < 0.05, **P < 0.01, ***P < 0.001, ****P < 0.0001 vs. IS. **d** Protein expression of NOX-1, *P < 0.05, **P < 0.01, ***P < 0.001, ****P < 0.0001 vs. IS. **e** Protein expression of NOX-2, *P < 0.05, **P < 0.01, ***P < 0.001, ****P < 0.0001 vs. IS. **f** Protein expression of p22phox, *P < 0.05, **P < 0.01, ***P < 0.001, ****P < 0.0001 vs. IS. **g** The oxidized protein expression, *P < 0.05, **P < 0.01, ***P < 0.001, ****P < 0.0001 vs. IS (Note: the right and left lanes shown on the upper panel represent protein molecular weight marker and control oxidized molecular protein standard, respectively). M.W. = molecular weight; DNP = 1–3 dinitrophenylhydrazone. All statistical analyses were performed by one-way ANOVA, followed by Bonferroni multiple-comparison post hoc test (*n* = 6 for each group). LCCA = left common carotid artery. SC = sham-operated control; IS = ischemic stroke (IS); EPC^3h^ = EPC administration from LCCA at 3 h after acute IS; EPC^3d^ = EPC administration from LCCA at day 3 after acute IS; EPC^7d^ = EPC administration from LCCA at day 7 after acute IS; EPC^14d^ = EPC administration from LCCA at day 14 after acute IS; EPC^28d^ = EPC administration from LCCA at day 28 after acute IS

The protein expressions of IL-1ß, TNF-α, NF-κB, and MMP-9, four indicators of inflammation, were lowest in SC and highest in IS, significant lower in EPC^3d^ and more significantly lower in EPC^3h^ than in EPC^7d^ to EPC^28d^, but they exhibited no difference among the EPC^7d^ to EPC^28d^ (Fig. 7).

**Fig. 8:**
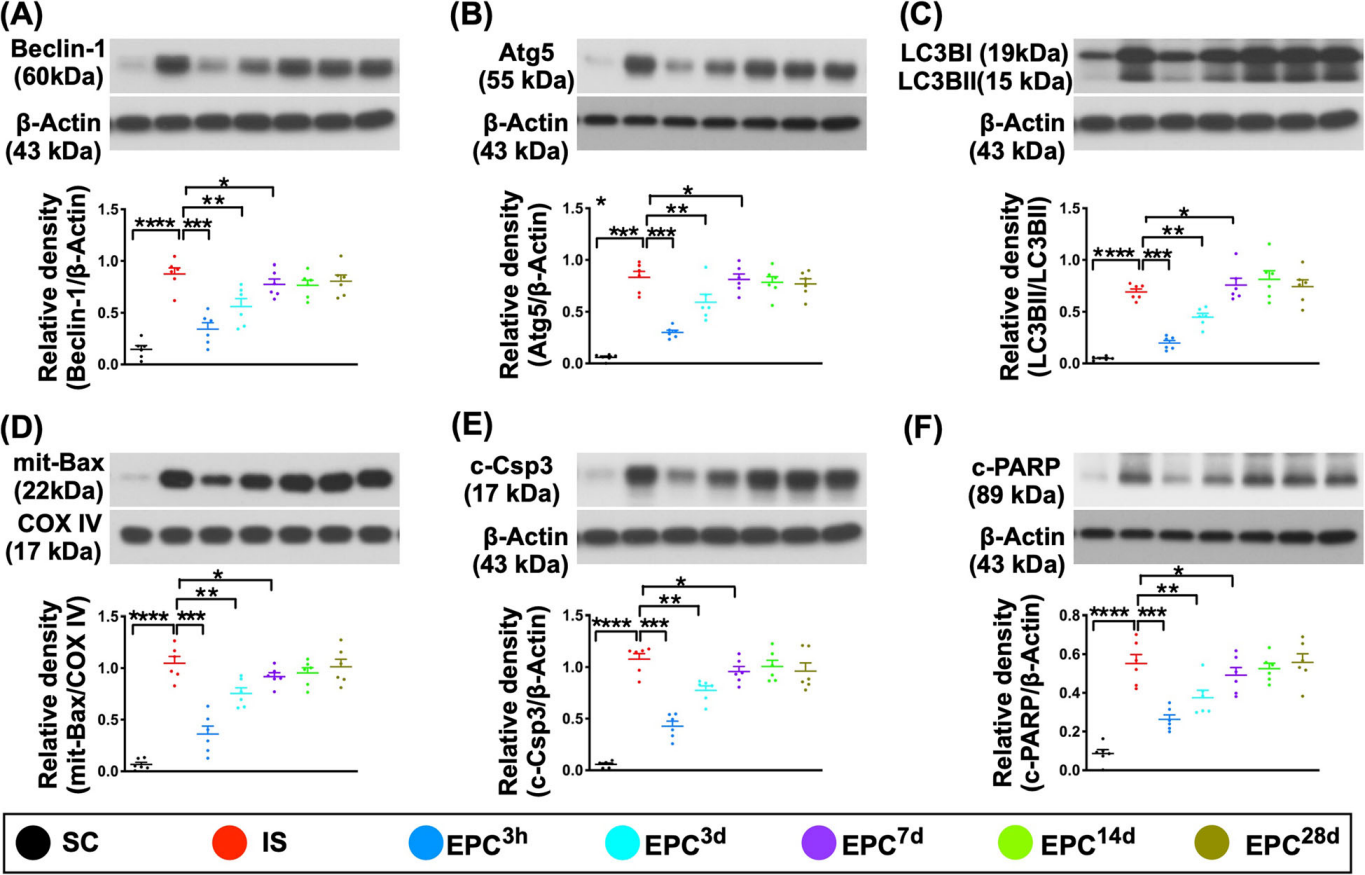
Protein expressions of autophagic and apoptotic biomarkers in brain infarct zone at day 60 after acute IS. **a** Protein expression of beclin-1, *P < 0.05, **P < 0.01, ***P < 0.001, ****P < 0.0001 vs. IS. **b** Protein expression of Atg-5, *P < 0.05, **P < 0.01, ***P < 0.001, ****P < 0.0001 vs. IS. **c** Protein expression of ratio of LC3B-II/LC3B-I, *P < 0.05, **P < 0.01, ***P < 0.001, ****P < 0.0001 vs. IS. **d** Protein expression of mitochondrial (mito)-Bax, *P < 0.05, **P < 0.01, ***P < 0.001, ****P < 0.0001 vs. IS. **e** Protein expression of cleaved caspase 3 (c-Csp3), *P < 0.05, **P < 0.01, ***P < 0.001, ****P < 0.0001 vs. IS. **f** Protein expression of cleaved Poly (ADP-ribose) polymerase (c-PARP), *P < 0.05, **P < 0.01, ***P < 0.001, ****P < 0.0001 vs. IS. All statistical analyses were performed by one-way ANOVA, followed by Bonferroni multiple-comparison post hoc test (*n* = 6 for each group). LCCA = left common carotid artery. SC = sham-operated control; IS = ischemic stroke (IS); EPC^3h^ = EPC administration from LCCA at 3 h after acute IS; EPC^3d^ = EPC administration from LCCA at day 3 after acute IS; EPC^7d^ = EPC administration from LCCA at day 7 after acute IS; EPC^14d^ = EPC administration from LCCA at day 14 after acute IS; EPC^28d^ = EPC administration from LCCA at day 28 after acute IS

Additionally, the protein expressions of NOX-1, NOX-2, oxidized protein, and p22phox, four indicators of oxidative stress, displayed an identical pattern of inflammation among the groups (Fig. 7). Furthermore, the protein expressions of Beclin-1, Atg-5, and ratio of LC3B-II/LC3B-I, three indicators of autophagic biomarkers, exhibited an identical pattern of inflammation among the groups (Fig. 8).

Moreover, the protein expressions of mitochondrial-Bax and cleaved PARP, two indices of apoptosis, showed an identical pattern to that of inflammation among the groups (Fig. 8).

Interestingly, previous study showed that cleaved caspase 3 expression was predominantly associated with cellular responses to stroke such as reactive astrogliosis and the infiltration of macrophages [20]. In the present study, we also found that the protein expression of cleaved caspase 3 was identical to the inflammation among the groups (Fig. 8), implicating that this parameter might not be necessarily a biomarker of apoptosis after IS.

### Inflammatory cell expressions of GFAP+ and microglial cells in BIA at day 60 after acute IS (Fig. 9)

The results of IF microscopic findings demonstrated that the expressions of GFAP and microglial cells, two indicators of inflammatory cells specifically situated in brain tissue, were lowest in SC, highest in IS, and significantly and progressively increased from EPC^3h^ to EPC^28d^, but it displayed no difference between EPC^7d^ and EPC^14d^.

**Fig. 9:**
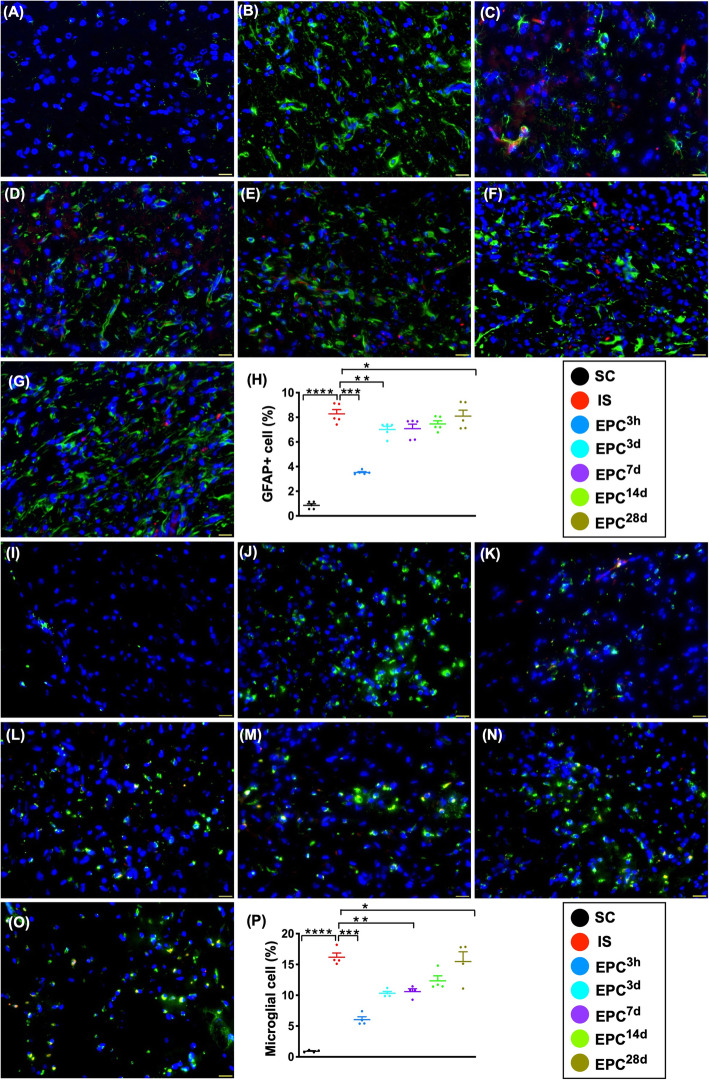
Inflammatory cell expressions in brain infarct zone at day 60 after acute IS. **a**–**g** Illustrating the immunofluorescent (IF) microscopic finding (× 400) for identification of positively stained glial fibrillary acidic protein (GFAP) cells (green color). Red color in **c** to **g** indicates the dye-stained implanted ECPs. **h** Analytical result of number of GFAP+ cells, *P < 0.05, **P < 0.01, ***P < 0.001, ****P < 0.0001 vs. IS. **i**–**o** Illustrating the IF microscopic finding (× 400) for identification of microglia+ cells (green color). Red color in **c** to **g** indicates the dye-stained implanted ECPs. **p** Analytical result of number of microglial cells, *P < 0.05, **P < 0.01, ***P < 0.001, ****P < 0.0001 vs. IS. Scale bars in the lower right corner represent 20 μm. All statistical analyses were performed by one-way ANOVA, followed by Bonferroni multiple-comparison post hoc test (*n* = 6 for each group). LCCA = left common carotid artery. SC = sham-operated control; IS = ischemic stroke (IS); EPC^3h^ = EPC administration from LCCA at 3 h after acute IS; EPC^3d^ = EPC administration from LCCA at day 3 after acute IS; EPC^7d^ = EPC administration from LCCA at day 7 after acute IS; EPC^14d^ = EPC administration from LCCA at day 14 after acute IS; EPC^28d^ = EPC administration from LCCA at day 28 after acute IS

## Discussion

This study which investigated the appropriate time point of intra-carotid administration of circulatory-derived autologous EPCs for improving the neurological function and reducing the BIV yielded several preclinical striking implications. First, despite the difference in the degree of preservation of neurological function and brain architecture, the EPC therapy at time intervals ≤ 14 days after IS still significantly preserved the neurological function and integrity of brain architecture in rat acute IS. Second, through different time intervals of administrating EPCs for rat after acute IS, this study identified that the optimal time point of EPC therapy for improving the neurological function and reducing the infarct zone was as early as possible (i.e., at 3 h after acute IS).

Intra-carotid administration of circulatory EPCs significantly improved neurological outcome and reduced the brain infarct size in rat after acute IS having been well recognized by our previous study [17]. Surprisingly, there is currently seldom data available to address the optimal timing of administration of EPCs after acute IS. In the current study, time courses of EPC therapy for identifying the most appropriate time point was done in rat IS model. The most important finding in the present study was that 3 h after acute IS was the best timing for EPC administration to improve the neurological function and preserve brain architecture. Additionally, our result also observed therapeutic window at ≤ 3 days upon cell administration also effectively preserved BIV and neurological function. Of importance was that all the parameters in molecular-cellular levels were consistent with the findings of brain MRI and corner test (i.e., neurological functional measurement). In this way, our findings clearly explained that EPC treatment was not only successful for the brain repair but also successful for the neuroprotection in rodent after acute IS. Accordingly, our finding, in addition to supporting the finding of our previous study [5], highlights the common concept that cell therapy should be performed “as early as possible” for ischemia-related organ dysfunction.

Interestingly, one previous study from Boltze et al. has demonstrated that administration of human umbilical cord blood mononuclear cells (hUCB MNCs) at 4, 24, or 72 h effectuated a significant improvement of functional defects in rodent [21]. Therefore, their results for the first time indicate a time window of therapeutic hUCB MNC application of at least 72 h. In this way, our findings were consistent with the findings from Boltze et al. [21].

Recently, we have performed a phase I clinical trial by utilizing the circulatory-derived autologous CD34+ cells to treat those old IS patients [18]. Interestingly, although the safety of the cell therapy was confirmed [18], the therapeutic impact on improving the neurological and recognizing outcomes regrettably left much to be desired, attributing mainly to late treatment. Some previous clinical trials have also demonstrated that cell therapy for patients after acute myocardial infarction (AMI) failed to improve the heart function and clinical outcome [22, 23]. In further, when we carefully analyzed the results from these clinical trials [22, 23], a commonality of late administration of stem cells to patients after AMI was uncovered. An essential finding in the present study was that as we serially tested the time points of administration of EPCs at 3 h and days 3, 7, 14, and 28 in rat after acute IS, the later the EPC administration, the lesser effective degree of neurological improvement was found in the present study. Accordingly, our finding, in addition to explaining why those clinical studies [18, 22, 23] failed to prove the effectiveness of stem cell therapy for the improvement of the left ventricular (LV) function in patients after AMI, supports the common concept that only early administration of stem cells is the fundamentally mechanistic basis for improving ischemia-related organs, especially in setting of acute IS and AMI.

The underlying mechanisms for improving the ischemia-related LV dysfunction after the cell therapy have been well recognized mainly due to the angiogenesis and neovascularization, chemokine/paracrine effects, stem cell homing, anti-inflammatory response, and immunomodulation, but they are less likely due to the implanted stem cells to differentiate into the cardiomyocytes [24–26]. Of these factors, the angiogenesis is one of the most important factors for improving the ischemia-related organ dysfunction [16, 17, 24–26]. An essential finding in the present study was that the molecular-cellular levels of angiogenesis parameters were markedly upregulated after IS stroke (i.e., due to an intrinsic response) and further markedly upregulated by EPC treatment, especially in the time point of early administration of EPCs to the animals after acute IS. Of particular importance was that increases of angiogenesis biomarker were significantly associated with improving neurological function and reducing the brain infarct zone in those animals receiving early EPC treatment. Our findings were consistent with those of previous studies [16, 17, 24–26].

A principal finding in the present study was that the inflammatory, oxidative stress, and apoptotic and autophagic biomarkers were substantially increased in acute IS animals than in those of SC animals. Additionally, the neurological impairment and brain infarct region were remarkably increased in these acute IS animals. Intriguingly, links between upregulations of inflammation, oxidative stress, apoptosis, and autophagic biomarkers and impaired ischemia-related organ dysfunction have been fully investigated by the previous experimental studies [17, 19, 24–28]. Along this line of thinking, our findings in addition to corroborating with the findings of previous studies [17, 19, 24–28], could, at least in part, explain why the neurological status and BIV were notably deteriorated in those of acute IS animals without treatment. However, these molecular-cellular perturbations were significantly reversed in IS animals after receiving the early EPC treatment, once again suggesting that EPC treatment in an appropriate time point could effectively improve outcome in setting of acute IS. Accordingly, our preclinical findings may provide clinically relevant information and encourage us to utilize EPC therapy for those acute IS patients, especially when they have poor response or are refractory to conventional therapy.

In the present study, we identified that EPC therapy for the rodent at 3 h after acute IS was the ideal time for preservation of BIV and neurological function. However, it is impractical to apply the autologous EPCs to the patients within 3 h after IS not only due to the time is not enough for the well preparation of EPCs but also due to there is a relatively high potential risk for the patients at this time point to receive the treatment. On the other hand, a previous study has demonstrated that cryopreservation of cells would reduce the efficacy [29]. Additionally, aging and senescence effects due to cryopreservation [30] could also contribute to negative or neutral results [31]. Accordingly, when hemopoietic-derived stem cell therapy is taken into consideration for acute IS patients, minimal manipulation (i.e., cells without cultivation) method to collect the circulatory-derived/bone marrow-derived autologous CD34+ cells would be more practical and suitable for the IS patients in our daily clinical practice.

### Study limitation

**Fig. 10:**
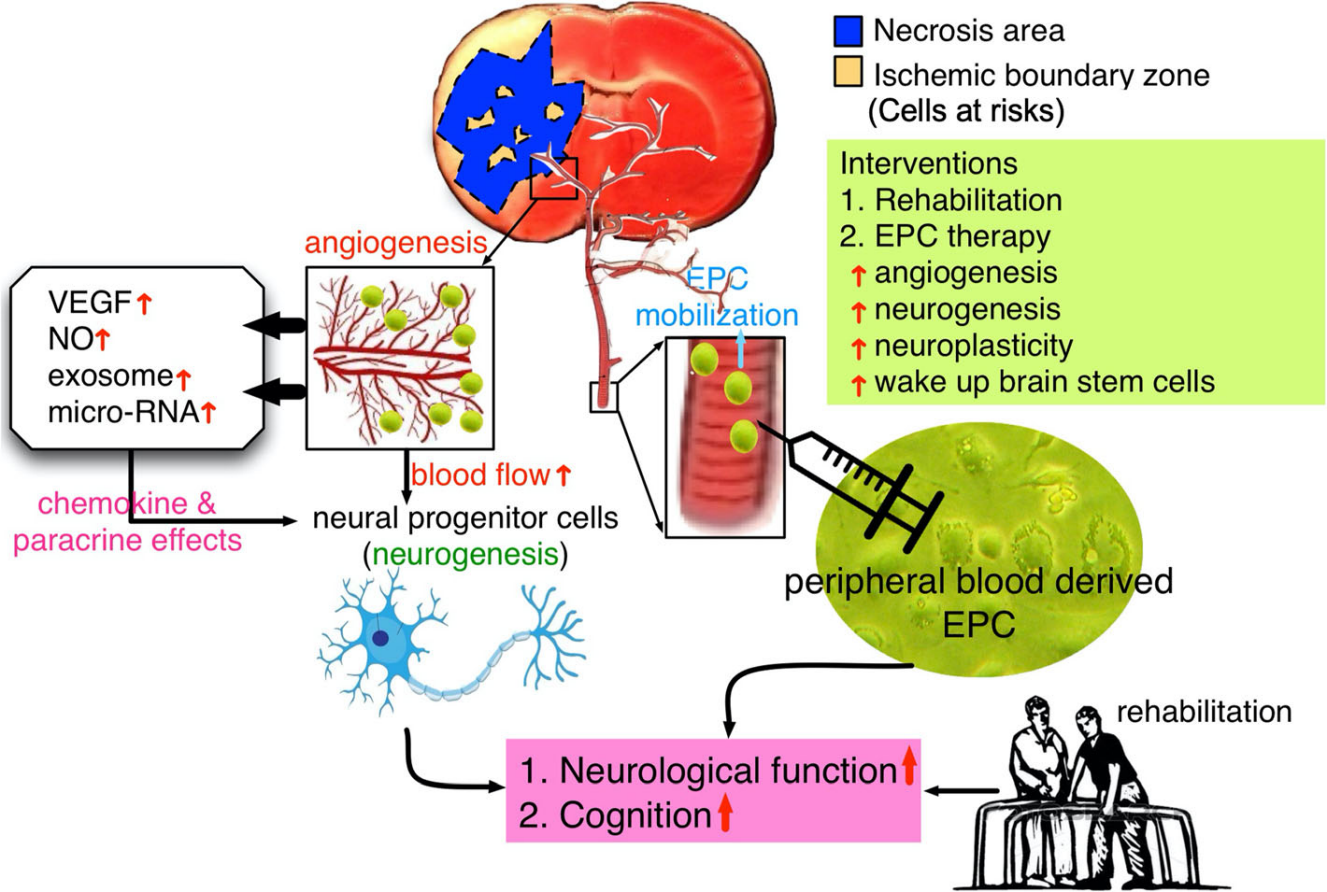
The underlying proposed mechanism of peripheral blood-derived endothelial progenitor cell (EPC) (i.e., CD34+ cells) therapy for improving the outcomes in patients or rodents after acute ischemic stroke. VEGF = vascular endothelial growth factors; NO = nitric oxide

Investigators have previously suggested that functional recovery after an experimental stroke should be assessed by multiple behavioral tests to prevent false-positive results [32]. In the present study, only a sensorimotor function (i.e., corner test) applied might not be optimal for long-term assessment of functional deficits in stroke, suggesting that selecting an appropriate task or a battery of tasks should be necessary in our study. Second, microembolisms and decreased cerebral blood flow during intra-arterial delivery of allogeneic bone marrow mesenchymal stem cells have been reported by a previous experimental study [33]. However, this study did not evaluate the possibility of microembolisms after EPC transplantation that could cause the complication of embolic events and concomitant new infarct zone. Third, there was a reduction of BIV in groups receiving cell therapy at days 14 and 28 by brain MRI finding. Generally, the infarct is fully developed around day 14, so any BIV reduction at EPC^28d^ group compared to that of the controls might be explained by (1) lesion size differences at baseline (2) less likely, any type of tissue regrowth, or(3) due to the angiogenesis and tissue regeneration in ischemic boundary zone (i.e., cell at risks) after EPC therapy (refer to Fig. 10, our proposed mechanism based on our findings). (4) One study have previously demonstrated that a principal role of CD34+ cell therapy in facilitating directly or indirectly an environment favorable for neovascularization of ischemic brain so that neuronal regeneration can proceed [34]. The result of this study [34] might also support the finding of our study. Finally, despite extensive works were done in the present study, the exactly underlying mechanisms of improving the outcomes after EPC therapy for acute IS remains uncertain. Based on the results of the present study, Fig. 10 provides the schematically proposed mechanism for the impact of EPC therapy on mammalians after acute IS.

In conclusion, the results of the present study proved that early administration of EPCs for acute IS rodent would provide the best effect on improving the neurological outcome and protecting the brain architecture against acute IS damage.

## Supplementary information

**Additional file 1.** Supplementary Figure 1 Illustrating the flow cytometric analysis for identification of the EPC surface markers (i.e., endothelial lineage) after 21-day cell culture. A) Illustrating the flow cytometric analysis for identification of EPC surface markers. B) Illustrating the morphologic feature (400x) of EPCs after 21-day cell culture, i.e., cobblestone-like morphology typical for endothelial cells. C) Expressed the data as mean ± SD (*n* = 4).

**Additional file 2.** Supplementary Figure 2 Schematic flow chart showing the time points of preparation of EPCs and the strategic management for the IS animals among the seven groups. EPC = endothelial progenitor cell; SC = sham-operated control.

## Data Availability

The data that support the findings of this study are available from the corresponding authors upon reasonable request.

## Abbreviations

AMI: Acute myocardial infarction
BIA: Brain infarct area
BIV: Brain infarct volume
DMEM: Dulbecco’s modified Eagle’s medium
ECL: Enhanced chemiluminescence
EPC: Endothelial progenitor cells
FBS: Fetal bovine serum
GFAP: Glial fibrillary acidic protein
HPFs: High-power fields
ICA: Intra-carotid arterial
IF: Immunofluorescent
IHC: Immunohistochemistry
IS: Ischemic stroke
LCCA: Left common carotid artery
LV: Left ventricular
MMP: Matrix metalloproteinase
MRI: Magnetic resonance imaging
PARP: *Poly (ADP-ribose) polymerase*
PVDF: Polyvinylidene difluoride
SDF-1α: Stromal cell-derived factor
SDS-PAGE: Sodium dodecyl sulfate-polyacrylamide gel electrophoresis
TBS: Tris-buffered saline
TE: Echo time
TR: Repetition time
VEGF: Vascular endothelial growth factor
vWF: von Willebrand factor
