# Supplementary figures and images for: Intra-carotid arterial transfusion of circulatory-derived autologous endothelial progenitor cells in rodent after ischemic stroke—evaluating the impact of therapeutic time points on prognostic outcomes
Source: Stem Cell Res Ther. 2020 Jun 5;11:219. doi: 10.1186/s13287-020-01739-y (PMC7275327; doi:10.1186/s13287-020-01739-y)

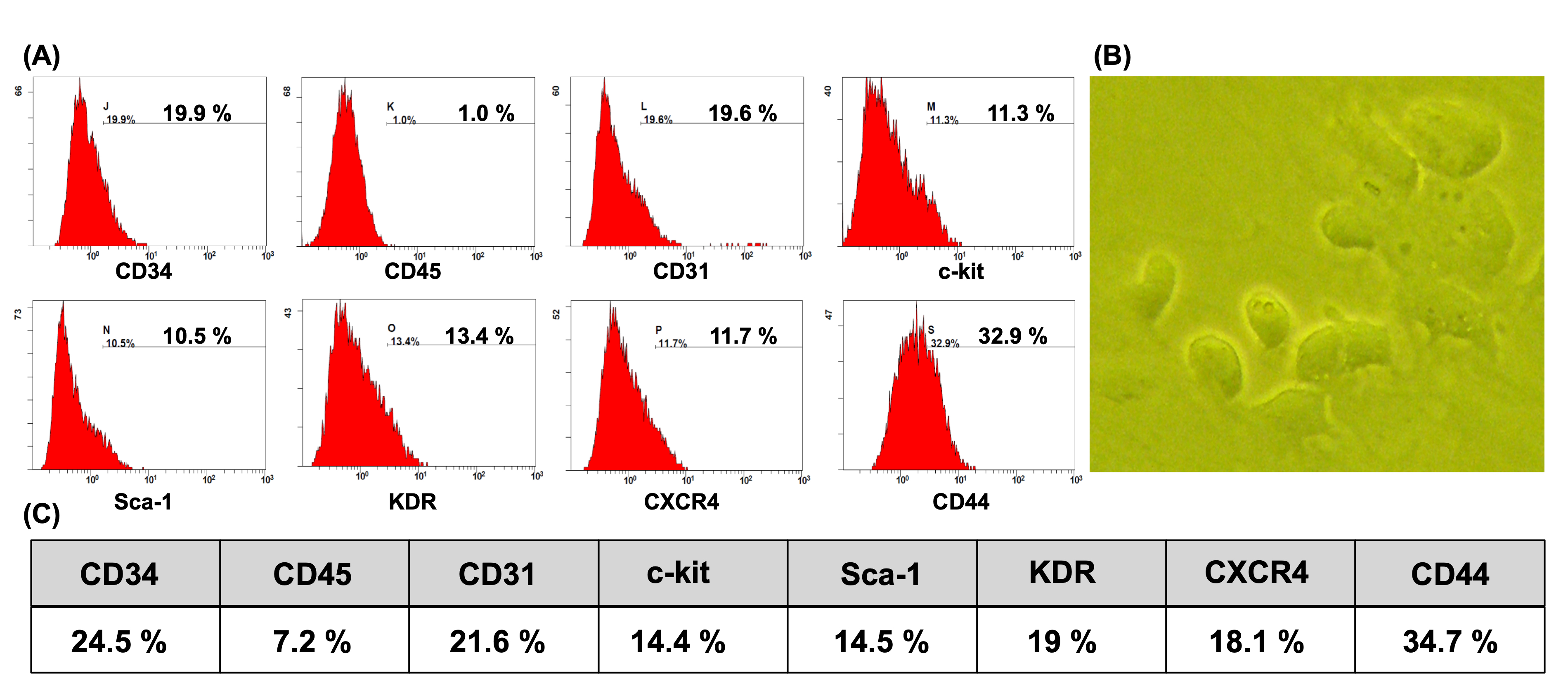

Supplement: Supplementary file 1 — Additional file 1. Supplementary Figure 1 Illustrating the flow cytometric analysis for identification of the EPC surface markers (i.e., endothelial lineage) after 21-day cell culture. A) Illustrating the flow cytometric analysis for identification of EPC surface markers. B) Illustrating the morphologic feature (400x) of EPCs after 21-day cell culture, i.e., cobblestone-like morphology typical for endothelial cells. C) Expressed the data as mean ± SD (n = 4). [file 13287_2020_1739_MOESM1_ESM.jpg]

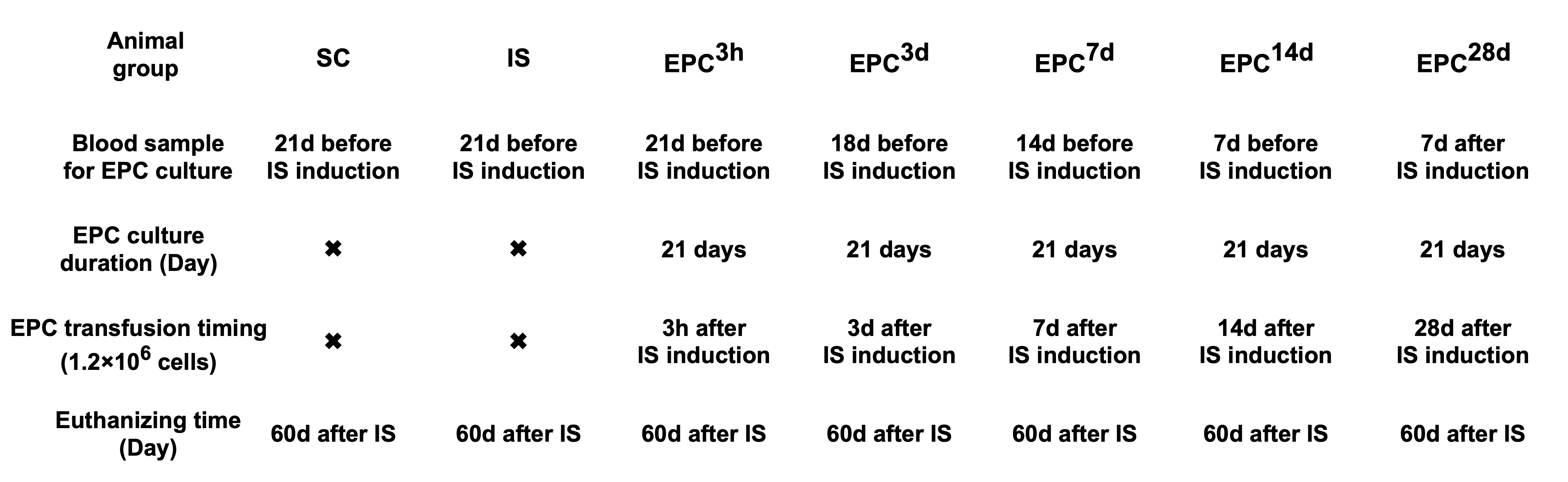

Supplement: Supplementary file 2 — Additional file 2. Supplementary Figure 2 Schematic flow chart showing the time points of preparation of EPCs and the strategic management for the IS animals among the seven groups. EPC = endothelial progenitor cell; SC = sham-operated control. [file 13287_2020_1739_MOESM2_ESM.jpg]
